# Biophysical and Biochemical Characterization of TP0037, a d-Lactate Dehydrogenase, Supports an Acetogenic Energy Conservation Pathway in Treponema pallidum

**DOI:** 10.1128/mBio.02249-20

**Published:** 2020-09-22

**Authors:** Ranjit K. Deka, Wei Z. Liu, Michael V. Norgard, Chad A. Brautigam

**Affiliations:** a Department of Microbiology, UT Southwestern Medical Center, Dallas, Texas, USA; b Department of Biophysics, UT Southwestern Medical Center, Dallas, Texas, USA; McGovern Medical School

**Keywords:** d-lactate dehydrogenase, *Treponema pallidum*, X-ray crystallography, acetogenesis, spirochetes

## Abstract

A longstanding conundrum in Treponema pallidum biology concerns how the spirochete generates sufficient energy to fulfill its complex pathogenesis processes during human syphilitic infection. For decades, it has been assumed that the bacterium relies solely on glucose catabolism (via glycolysis) for generation of its ATP. However, the organism’s robust motility, believed to be essential for human tissue invasion and dissemination, would require abundant ATP likely not provided by the parsimony of glycolysis. As such, additional ATP generation, either via a chemiosmotic gradient, substrate-level phosphorylation, or both, likely exists in T. pallidum. Along these lines, we have hypothesized that T. pallidum exploits an acetogenic energy conservation pathway that relies on the redox chemistry of flavins. Central to this hypothesis is the apparent existence in T. pallidum of an acetogenic pathway for the conversion of d-lactate to acetate. Herein we have characterized the structural, biophysical, and biochemical properties of the first enzyme (d-lactate dehydrogenase [d-LDH]; TP0037) predicted in this pathway. Binding and enzymatic studies showed that recombinant TP0037 consumed d-lactate and NAD^+^ to produce pyruvate and NADH. The crystal structure of TP0037 revealed a fold similar to that of other d-acid dehydrogenases; residues in the cofactor-binding and active sites were homologous to those of other known d-LDHs. The crystal structure and solution biophysical experiments revealed the protein’s propensity to dimerize, akin to other d-LDHs. This study is the first to elucidate the enzymatic properties of T. pallidum’s d-LDH, thereby providing new compelling evidence for a flavin-dependent acetogenic energy conservation (ATP-generating) pathway in T. pallidum.

## Abstract (precis)

Because T. pallidum lacks a Krebs cycle and the capability for oxidative phosphorylation, historically it has been difficult to reconcile how the syphilis spirochete generates sufficient ATP to fulfill its energy needs, particularly for its robust motility, solely from glycolysis. We have postulated the existence in T. pallidum of a flavin-dependent acetogenic energy conservation pathway that would generate additional ATP for T. pallidum bioenergetics. In the proposed acetogenic pathway, first d-lactate would be converted to pyruvate. Pyruvate would then be metabolized to acetate in three additional steps, with ATP being generated via substrate-level phosphorylation. This study provides structural, biochemical, and biophysical evidence for the first T. pallidum enzyme in the pathway (TP0037; d-lactate dehydrogenase) requisite for the conversion of d-lactate to pyruvate. The findings represent the first experimental evidence to support a role for an acetogenic energy conservation pathway that would contribute to nonglycolytic ATP production in T. pallidum.

## INTRODUCTION

Syphilis is a chronic, complex, sexually transmitted infection of humans caused by the spirochetal bacterium Treponema pallidum subsp. *pallidum*. Despite decades of intensive efforts toward its epidemiological control, syphilis continues to play a prominent role worldwide (1–7). Incidence increases in the United States (8–12) have been particularly disturbing given the serious medical implications of syphilis as a chronic, progressive infection, coupled with its added threats of congenital syphilis and syphilis as a cofactor for the bidirectional transmission of HIV. These disturbing trends underscore the importance of continued efforts to elucidate the complex parasitic strategy of T. pallidum, with the goals of developing new ways to thwart infection using interventions based on a more complete understanding of syphilis pathogenesis.

Although syphilis is one of the oldest recognized sexually transmitted infections of antiquity (13, 14), T. pallidum is among the most poorly understood of all human bacterial pathogens. This is because research on T. pallidum historically has been hampered by enormous experimental constraints, most notably (i) the inability to cultivate the organism continuously *in vitro* (15), (ii) the consequent lack of a genetic manipulation system (mutant analysis not possible), and (iii) the atypical/noncanonical nature of many of the spirochete’s proteome components, often rendering them refractory to predictive analysis via contemporary bioinformatics. Only recently has an improved method for the sustained cultivation of T. pallidum *in vitro* been achieved (coculture with rabbit epithelial cells) (16), but it remains to be determined whether this promising new development will lead to substantive advances.

As an approach to circumvent historic research barriers and to elucidate previously unappreciated molecular and mechanistic features of T. pallidum, we have focused on discerning salient structure-function relationships for T. pallidum’s membrane lipoproteins (LPs) (7, 17–31). Membrane LPs serve many key functions in pathogenic bacteria (32, 33), and numerous studies point to the importance of LPs in crucial aspects of T. pallidum biology (about 4.4% of T. pallidum’s genome encodes 48 or more LPs) (34, 35). Our discovery-driven approach has involved characterizing recombinant versions of the treponemal LPs using structural biology, protein biophysics, and biochemistry (17–31, 36). These efforts have led to a number of novel discoveries for T. pallidum (17, 21–23, 25–28, 30), some of which are applicable to other bacteria (22, 23, 25, 27–29, 36–39). Particularly noteworthy is our discovery of an ABC-type riboflavin uptake system (27) and elucidation of dual enzymatic activities for TP0796 (also known as Ftp) as a novel flavin adenine dinucleotide (FAD) pyrophosphatase/flavin mononucleotide (FMN) transferase (21, 28). These advances, in turn, led us to identify a periplasmic posttranslational protein FMNylation pathway in T. pallidum that likely modulates intracellular flavin homeostasis for key enzymatic and cellular redox reactions (21, 27, 28). The combined observations have prompted us to propose that T. pallidum relies on a “flavin-centric” metabolic lifestyle for parasitism of its obligate human host (21).

**FIG 1:**
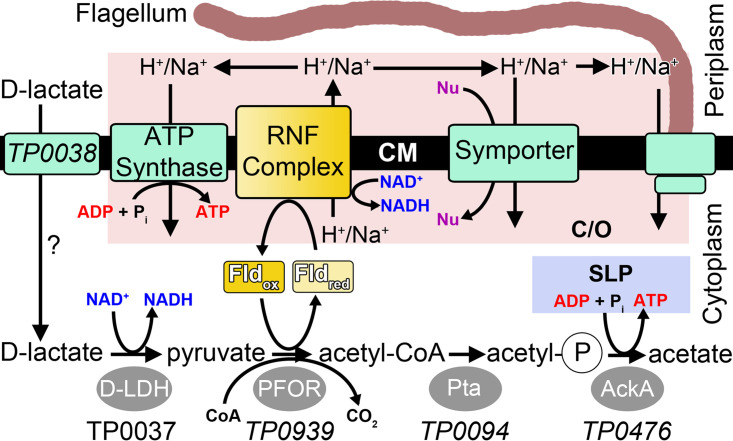
Enzymology and bioenergetics of the putative acetogenic pathway in T. pallidum. The metabolites of the pathway are shown near the bottom of the figure. Enzymes are gray ovals. Membrane proteins are green rectangles. The yellow rectangles are flavoproteins. The speculative role for TP0038 in d-lactate import is denoted by a question mark next to the arrow indicating transport. The substrate-level phosphorylation in the acetogenic pathway is labeled SLP and is enclosed in a blue box. Reactions utilizing or generating the chemiosmotic gradient are in a pink box labeled C/O. TP0037 is identified as the d-LDH in this study, and the names of the gene products that putatively fulfill the enzymatic functions in the acetogenic pathway or d-lactose transport are shown in italics. Abbreviations: d-LDH, d-lactate dehydrogenase; PFOR, pyruvate:flavodoxin oxidoreductase; Pta, phosphate acetyltransferase; AckA, acetate kinase; Fld, flavodoxin; CM, cytoplasmic membrane; P_i_, inorganic phosphate; acetyl-P, acetyl phosphate; Nu, nutrient.

A theme that has emerged from our structure-function studies is that T. pallidum encounters numerous essential host-derived nutrients and exploits many of its periplasmic membrane LPs as components of transport systems (17, 20, 25, 26, 30). As such, T. pallidum utilizes its membrane LPs to engage a number of downstream metabolic pathways as part of its flavin-centric metabolic lifestyle, engendering an inextricable metabolic linkage between the periplasmic (external) and cytoplasmic (internal) cellular compartments. This includes a cytoplasmic flavin salvage pathway (7, 27) and likely an atypical flavin-dependent *Rhodobacter* nitrogen fixation (RNF)-type energy conservation system (7, 21) (Fig. 1).

Historically, it has been believed that T. pallidum fulfills its energy requirements solely from the glycolytic processing of glucose (7, 34, 40) because utilization of other organic substrates as alternate energy or carbon sources has remained unclear (7). However, the minimal yield of ATP from glycolysis is seemingly inconsistent with the organism’s need for abundant ATP, not only for its extensive repertoire of ATP-dependent nutrient uptake transporters but also to satisfy its robust motility (7, 41, 42) and for other cellular demands. To address this conundrum, we have proposed the existence in T. pallidum of a flavin-dependent RNF redox pump (to generate a chemiosmotic gradient) (21) that would contribute to ATP synthesis via energizing a cognate ATP synthase module (Fig. 1) (7, 21, 27). Finally, ATP could also be generated via substrate-level phosphorylation from the conversion of d-lactate to acetate via acetogenesis (Fig. 1). In the acetogenesis pathway (43) (Fig. 1), pyruvate, a key pathway intermediate, is converted to acetyl coenzyme A (acetyl-CoA) via the flavin-dependent pyruvate flavodoxin oxidoreductase (PFOR); this reaction yields reduced flavodoxin (central electron carrier), from which an electron is transferred (via the flavin moiety) to energize the flavin-based RNF complex for additional ATP production (7, 21).

The existence of a flavin-based energy conservation pathway in T. pallidum has been predicted by our previous *in silico* analysis (7, 21). However, the prospect of an acetogenic energy conservation pathway in T. pallidum has remained uncertain due to a lack of corroborating experimental evidence. If T. pallidum is indeed able to carry out acetogenesis (Fig. 1), then the critical first step in the pathway would be the oxidation of d-lactate to pyruvate via d-lactate dehydrogenase (using NAD^+^ as a cofactor) (7, 43). In this regard, the gene product of *tp0037* is annotated as a d-lactate dehydrogenase (d-LDH) (7, 34). We crystallized recombinant TP0037, determined its X-ray crystal structure at a resolution of 1.38 Å, and performed additional biophysical and biochemical studies to characterize the protein. Our work now provides compelling evidence in support of a flavin-based d-lactate acetogenic energy conservation pathway in T. pallidum; acetogenesis would not only contribute to nonglycolytic ATP generation in T. pallidum but also serve cellular redox and bioenergetics. Our findings also help to explain the enigmatic metabolic adaptation(s) exploited by the pathogenic spirochete as it disseminates under low oxygen tension and encounters changes in external nutrient sources as part of its stealth pathogenicity. The possible origin of d-lactate as a substrate for the acetogenic energy conservation pathway in T. pallidum is discussed.

## RESULTS

### Crystal structure of TP0037.

We hyperexpressed full-length TP0037 and purified it to apparent homogeneity. The protein yielded crystals that diffracted well, and molecular-replacement phasing followed by standard refinement approaches resulted in a model for the protein at a resolution at 1.38 Å. All validation and data quality statistics indicated that the data and the model were of high quality (Table 1). The difference electron-density maps associated with this structure were carefully scrutinized for evidence of any bound substrates or cofactors, but the only convincing densities were assigned to solutes in the crystallization medium (ethylene glycol and chloride ion). We hence considered the structure to be the unliganded or an “apo” form of the protein.

**TABLE 1:** Data collection and refinement statistics of TP0037 crystals

Parameter	Value(s)
PDB accession no.	7JP2
Data collection	
Space group	P2_1_2_1_2_1_
Unit cell dimensions (Å)	
* a*	72.7
* b*	94.7
* c*	101.4
α (°)	90
β (°)	90
γ (°)	90
Resolution (Å)	38.0–1.38 (1.40–1.38)^*a*^
Completeness (%)	99.5 (98.8)
Multiplicity	4.3 (4.3)
No. of unique reflections	143,109 (7,071)
* R*_merge_^*b*^	0.047 (0.296)
<*I*>/`ι>σ_I_`/ι>	21.1 (3.3)
Wilson *B* (Å^2^)	8.3
Refinement	
Resolution (Å)	38.1–1.38
No. of residues	661
No. of nonsolvent atoms	50
No. of solvent atoms	643
Maximum likelihood coordinate error (Å)	0.09
Average *B*-factors	
Macromolecule (Å^2^)	12.69
Solvent (Å^2^)	22.83
*R* values	
* R*_work_^*c*^	0.136
* R*_free_^*d*^	0.158
Ramachandran statistics	
Outliers (%)	0.3
Most favored region (%)	98.3
RMSD	
Bonds (Å)	0.005
Angles (°)	0.9

a Numbers in parentheses are the values reported for the highest-resolution shell of reflections.

b $R_{\text{merge}}=\sum_{\mathrm{hkl}}\sum_{i}\left|I_{h,i}-〈I_{h}〉\right|/\sum_{\mathrm{hkl}}\sum_{i}I_{h,i}$ where the outer sum (*hkl*) is over the unique reflections and the inner sum (*i*) is over the set of independent observations of each unique reflection.

c $R_{\text{work}}=\sum_{\mathrm{hkl}}\left|\left|F_{o}\right|-\left|F_{c}\right|\right|/\sum_{\mathrm{hkl}}\left|F_{o}\right|$, where *F_o_* and *F_c_* are observed and calculated structure factor amplitudes, respectively.

d *R*_free_ is calculated using the same formula as *R*_work_, but the set *hkl* is a randomly selected subset (5%) of the total structure factors that are never used in refinement.

**FIG 2:**
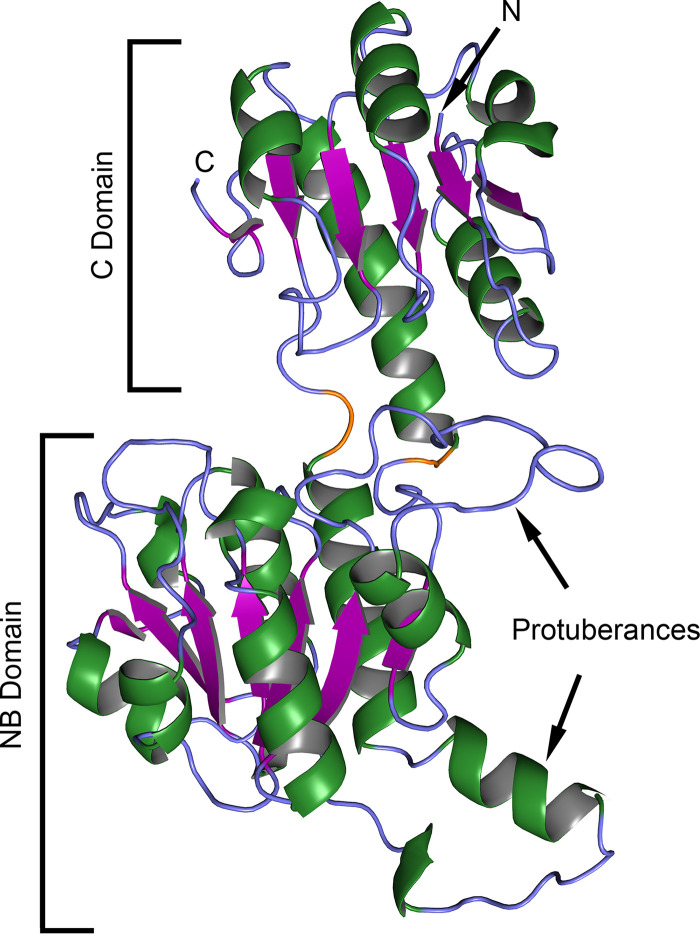
Crystal structure of TP0037. The crystal structure of TP0037 is shown in ribbon representation, with helical elements colored green, strands purple, and regions with no regular secondary structure light blue. The two connector regions noted in the text are colored orange. The N and C termini are labeled “N” and “C,” respectively. Other regions noted in the text are annotated.

The TP0037 crystal structure featured two domains with a cleft between them (Fig. 2; see also Fig. S1 in the supplemental material). The smaller domain which, by convention (44), we termed the “C” domain (“C” being an abbreviation for “catalytic”), comprised a central β-sheet flanked by α-helices. The sheet was composed of five parallel strands and one antiparallel one. Both the amino and carboxyl termini were present in the C domain.

10.1128/mBio.02249-20.1 FIG S1 Topological organization of TP0037. The overall organization of TP0037 into secondary structural units is shown, with the orientation similar to that presented in Fig. 2 of the main text. β-Strands are shown as purple arrows, and α-helices or 3_10_-helices are shown as green rectangles. Helices are associated with the two β-sheets shown, one in the NB domain, one in the C domain, and thus light green helices are below the plane of the respective sheet, while dark green ones are above the plane of the sheet. The labeling was done according to conventions for 2-hydroxyacid dehydrogenases (82). All secondary structural elements are shown roughly to scale, but connections are not, especially in the hinge region, which is elongated to achieve separation between the domains. Download FIG S1, TIF file, 0.5 MB. Copyright © 2020 Deka et al. 2020 Deka et al. This content is distributed under the terms of the Creative Commons Attribution 4.0 International license.

**FIG 3:**
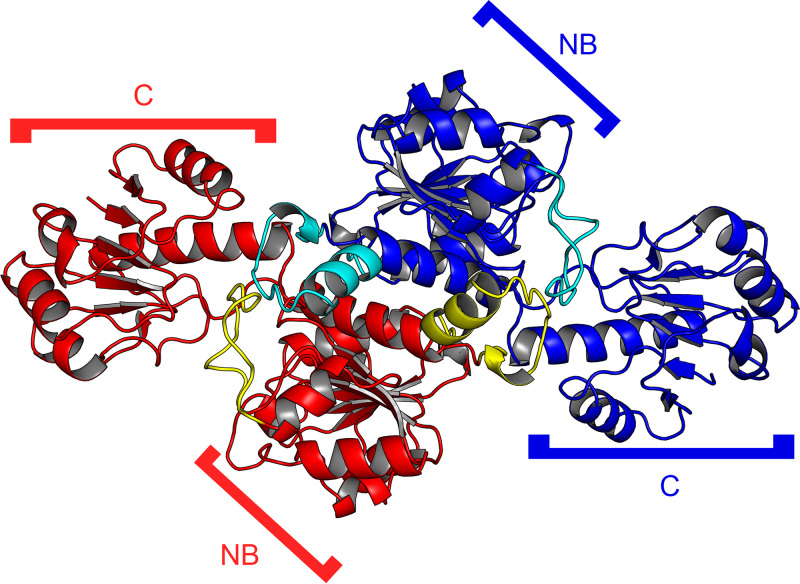
The dimer of TP0037 in the asymmetric unit of the crystals. Ribbon representations of the two monomers found in the asymmetric unit of the TP0037 crystals are shown. One monomer is colored blue, the other red. Protuberances belonging to the blue monomer are colored cyan, and those belonging to the red monomer are colored yellow. The NB and C domains noted in Fig. 2 are also labeled here.

The second, larger domain, termed the NB domain (NB for “nucleotide-binding”) because of its role in binding the nucleotide-based cofactor NAD^+^ (or NADH) (44), harbored a centrally located seven-strand parallel β-sheet. Again, α-helices decorated both faces of the sheet. Two prominent protuberances emanated from the core of this domain were defined in the primary structure by residues 119 to 141 and 264 to 279 (Fig. 2), and they play roles in the dimer interface (see below, Fig. 3).

The two domains were connected by a small region comprising the two points at which the main chain crossed from one domain to the other. They are defined by residues 300 and 301 and residues 101 and 102 (Fig. 2). Because interdomain motion is apparently a hallmark of the catalytic action of this class of proteins (44, 45), we deemed this part of the protein the “hinge region.” This portion of the protein and the protuberances noted above do not exhibit any evidence of dynamic or static disorder, as the *B*-factors for their respective C_α_ atoms do not range much above the average *B*-factor for all atoms in the protein.

Two of these bilobed monomers were found in the asymmetric unit of the TP0037 crystals (Fig. 3). The interface between the monomers was formed almost entirely by the respective NB domains such that the two C domains were found on opposite ends of the dimer. The NB domain protuberances noted above figured prominently in the intermolecular contacts, reaching across the dimeric interface and interacting with each other (Fig. 3). According to the PISA web server (46), the observed dimer had 5,480 Å^2^ of buried surface area, and the dissociation of the dimer was calculated to have a free dissociation energy (Δ*G*_diss_) of +36.1 kcal/mol, suggesting that the dimer is stable in solution.

### Comparisons to other structures.

Three-dimensional structural comparisons to known protein structures were conducted using either secondary-structure matching (SSM) (47) or a heuristic approach (DALI) (48). Both algorithms found that TP0037 best matched the structure of a putative d-lactate dehydrogenase from a *Sporolactobacillus* species (PDB accession number 4XKJ [49]). The root mean square deviation (RMSD) over 332 matched C_α_ atoms was 1.63 Å for SSM, and it was 1.7 Å for the 332 matched C_α_ atoms by DALI. The second-best match is the d-lactate dehydrogenase from Lactobacillus bulgaricus (PDB accession numbers 1J4A and 1J49 [45, 50, 51]; referred to as LbD-LDH hereafter), which has been shown to have d-lactate dehydrogenase activity *in vitro* (52). SSM ranked 1J4A (50) as a better match to TP0037, with an RMSD of 1.68 Å over 331 matched C_α_ atoms, while DALI gave preference to the related entry 1J49 (51), comparing it to TP0037 with a 1.9-Å RMSD over 332 C_α_ atoms. The *Sporolactobacillus* and L. bulgaricus d-LDHs have amino acid sequences that are 38% identical and that are structurally similar, having a 1.7-Å RMSD over 331 matched C_α_ atoms. Other high-ranking structural matches to TP0037 included additional d-isomer-specific 2-hydroxyacid dehydrogenases from other bacterial species, including Lactobacillus jensenii and Acidaminococcus fermentans.

The crystal structure of the bona fide d-lactate dehydrogenase from LbD-LDH was determined with NADH bound (this structure has the accession code IJ49, referred to above) (45). We superposed this structure on that of TP0037, with the goal of examining the residues from LbD-LDH with side chains in contact with NADH and their structural homologs in TP0037. Although conformational variability is a hallmark of d-LDHs, no significant conformational differences were observed between TP0037 and NADH-bound LbD-LDH, as implied by the low pairwise RMSD (see above). In LbD-LDH, NADH bound to the portion of the NB domain that faced the interdomain cleft. In essentially all cases, the superposition showed that LbD-LDH residues in contact with the NADH were matched by structurally homologous side chains from TP0037 having either identical or similar chemical natures (Table 2). It therefore appeared likely that TP0037 was capable of binding to NADH in an analogous manner to LbD-LDH.

**TABLE 2:** Residues from *L. bulgaricus* d-LDH with side chains in contact with NADH and structural homologs from T. pallidum TP0037

*L. bulgaricus* residue	T. pallidum structural homolog residue
Y101	Y101
I106	I106
V151	L152
G152	G153
G154	G155
H155	R156
I156	I157
Y174	F175
D175	D176
I176	P177
V206	M206
P207	P207
N212	S212
V233	T233
D259	D259
H296	H296
A298	A298
F299	F299

**FIG 4:**
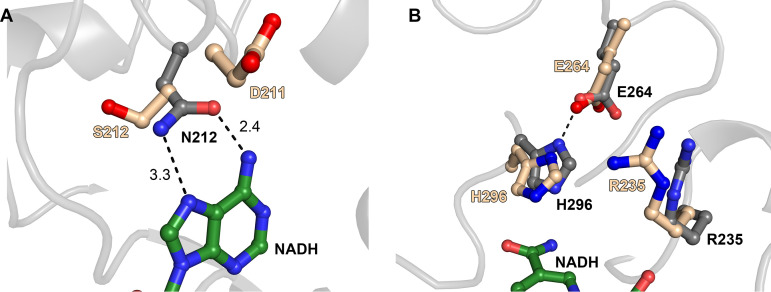
Homologous residues in the cofactor-binding sites and active sites of LbD-LDH and TP0037. In both parts, gray carbon atoms denote that they are from the LbD-LDH structure, and tan carbon atoms are from the TP0037 structure. Green carbon atoms are shown for the NADH bound to the LbD-LDH structure. Nitrogen atoms are colored blue, and oxygen atoms red. Secondary structure from LbD-LDH is shown in light gray for clarity. Black dashes are putative hydrogen bonds. (A) A difference in NADH binding. The distances in ångströms are shown next to the broken lines. (B) Key residues in the respective active sites. The position of the nicotinamide ring in the LbD-LDH structure is shown for reference.

A notable exception to the chemical correspondence between the NADH-binding residues of LbD-LDH and the putative ones of TP0037 occurred at contacts between LbD-LDH and the adenine moiety of NADH (Fig. 4A). LbD-LDH possesses an asparagine residue (N212) that apparently made bidentate contacts with the two nitrogen atoms on the Hoogsteen edge of the base. However, the corresponding residue in TP0037 is S212, which is incapable of the same bidentate interaction. Several possibilities for the interaction of TP0037 with this part of the cofactor emerged from a close examination of the superposition. First, S212 may contact the adenine ring with a single hydrogen bond to either N7 or the exocyclic N6 amino group; a bifurcated hydrogen bond to them both was also possible. Also, the preceding amino acid in TP0037 is D211 (A211 in LbD-LDH), which could plausibly adopt a position to interact with N6, with S212 contacting N7. Although the existence of P177 (TP0037) in the place of LbD-LDH’s I176 (Table 2) may appear to be a significant change, these residues exist in nontopologically constrained loop regions, and the chemical characters of the respective side chains (containing methylene and/or methyl groups) are similar, and thus, the difference here is not expected to hamper NADH binding. Attempts to resolve these issues by cocrystallizing TP0037 with NADH or NAD^+^ did not yield crystals suitable for structural characterization.

Superpositioning also afforded us the opportunity to scrutinize the disposition of the residues responsible for catalysis in LbD-LDH and their structural homologs in TP0037. In LbD-LDH, these residues were E264, H296, and R235. The side chain carboxylate group of E264 was in contact with the imidazolium moiety of H296, with the guanidinium group of R235 poised nearby (Fig. 4B). E264, H296, and R235 were the structural homologs of these amino acids in TP0037, and they adopted similar relative positions in the putative active site of the treponemal protein. Hence, the structure of TP0037 revealed that parts of the interdomain cleft closely resembled both the cofactor-binding site and the active site of a known d-lactate dehydrogenase (LbD-LDH).

### Biophysical studies of TP0037.

d-Lactate dehydrogenases are thought to exist as homodimers in solution, with no indication of ligand-induced oligomerization. However, this supposition has been inferred mostly from crystal structures (44, 45), size exclusion chromatography (53, 54), and native polyacrylamide gel electrophoresis (55). In this study, we sought independent verification of the dimeric oligomerization state of TP0037 by characterizing its solution behavior using dynamic light scattering (DLS), static light scattering (SLS), and analytical ultracentrifugation in the sedimentation velocity mode (SV).

**FIG 5:**
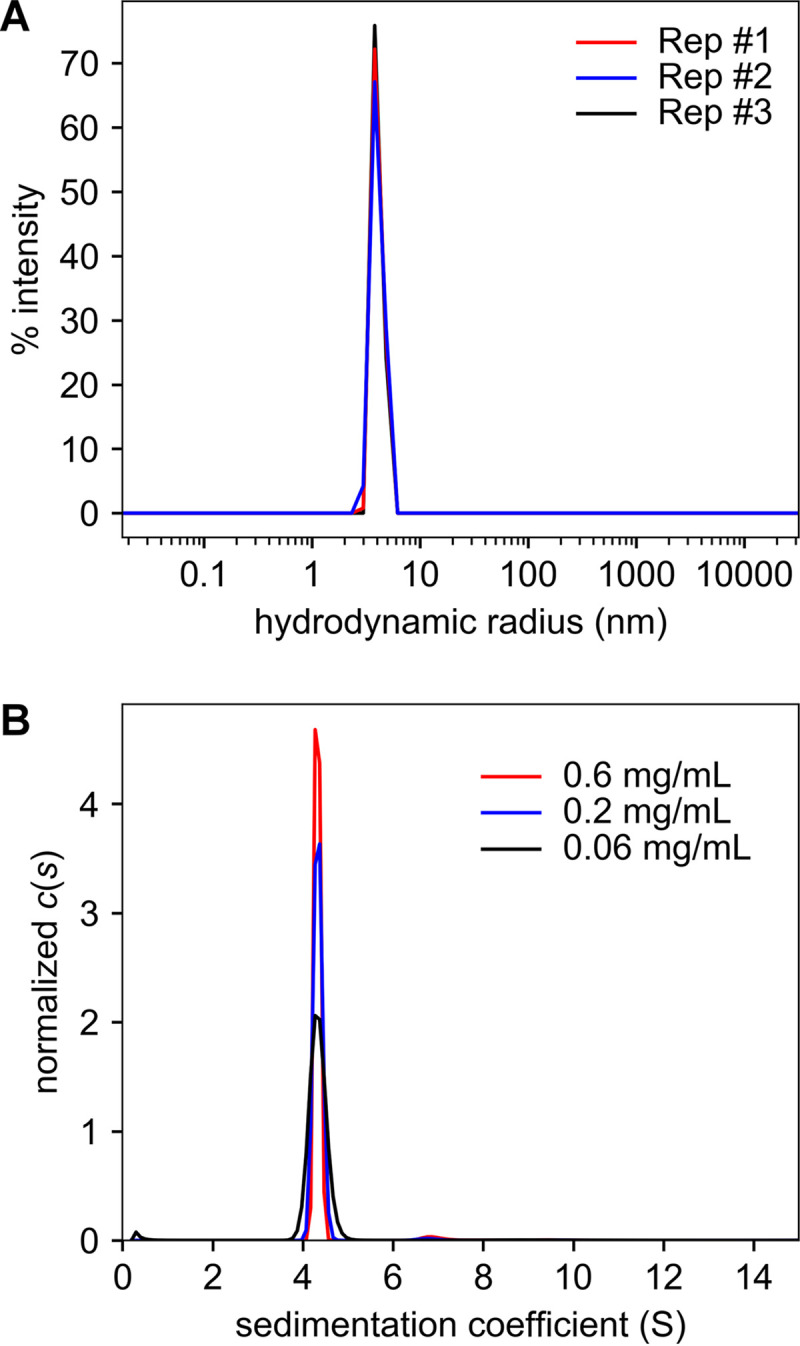
Hydrodynamics of TP0037. (A) Hydrodynamic radius distributions of TP0037. Three technical replicates (Rep #1 to Rep #3) are shown. (B) Sedimentation coefficient [*c*(*s*)] distributions for TP0037. Distributions for three different concentrations are shown. Both parts were rendered using GUSSI (94).

DLS experiments revealed that the protein was essentially monodisperse (Fig. 5A). The average polydispersity index calculated from the light-scattering data was very low (0.01 ± 0.02). The intensity-averaged hydrodynamic radius calculated from these experiments was 3.94 ± 0.08 nm. The low polydispersity of the protein enabled an accurate determination of its molar mass using SLS. That calculation yielded a molar mass of 78,100 ± 100 g/mol. With a monomeric molar mass of 39,190 g/mol, these data were consistent with TP0037 existing as a homodimer in solution.

As an orthogonal method to examine both the monodispersity and the molar mass of TP0037, we also performed SV experiments and evaluated the data using the *c*(*s*) methodology (56, 57). These studies recapitulated the high degree of monodispersity in our TP0037 preparations, as the *c*(*s*) distributions were dominated by a single peak at 4.3 Svedberg units (S) (Fig. 5B). No propensity for dissociation (smaller species) nor additional association (larger species) was noted over a 10-fold span of the concentration of TP0037 (Fig. 5B). The average molar mass calculated from the three experiments was 75,800 ± 1,600 g/mol, again consistent with the existence of TP0037 as a dimer in solution.

We also assessed ligand binding to TP0037 using differential scanning fluorimetry (DSF) (Table 3) (58). We found that the presence of 5 mM NAD^+^ raised the melting temperature of TP0037 by approximately 4°C (*P* = 1.7 × 10^−7^, one-sided *t* test). Incubating 20 mM d-lactate (without NAD^+^) with the protein raised the melting temperature (*T_m_*) more modestly (ca. 1°C) but still significantly (*P* = 0.0002, one-sided *t* test). l-lactate raised the temperature less (ca. 0.2°C) but measurably (*P* = 0.04, one-sided *t* test). These results confirm the binding of the necessary cofactor for catalysis and provide evidence for a strong preference to act on d-lactate rather than l-lactate.

**TABLE 3:** *T_m_* values for TP0037 with or without ligands present

TP0037 protein	*T_m_* value (°C)^*a*^ for TP0037 with the following additive:			
	No additive	5 mM NAD^+^	20 mM d-lactate	20 mM l-lactate
Wild-type	49.10 ± 0.16	53.38 ± 0.09	50.12 ± 0.07	49.336 ± 0.011
Y101A	53.48 ± 0.06	56.97 ± 0.05	53.79 ± 0.06	53.48 ± 0.03
R235A	55.85 ± 0.03	59.41 ± 0.05	56.207 ± 0.016	55.91 ± 0.02
H296A	48.68 ± 0.07	51.69 ± 0.17	48.810 ±0.018	48.71 ± 0.06

a All results are the means ± standard deviations from three separate *T_m_* determinations.

Mutations of the putative active site residues Y101, R235, and H296 to alanine were made in anticipation of testing their respective activities (see the next section). We noted the same overall pattern on the respective *T_m_* values, i.e., 5 mM NAD^+^ raised the *T_m_* the most, with d-lactate showing a higher increase than l-lactate. We also observed that two of the mutants, Y101A and R235A, resulted in significant rises in *T_m_* with no additives (4.4°C and 6.7°C, respectively, with respective *P* values of 1.2 × 10^−6^ and 1.5 × 10^−6^ in one-sided *t* tests). The origins of these substantial gains in stability are unknown but clearly indicate that these residues contribute to the metastability of the protein.

### Enzyme activity of TP0037.

To test whether TP0037 had d-lactate dehydrogenase activity, we performed an *in vitro* enzyme assay using the purified, monodisperse protein (Table 4). The assay utilized a colorimetric/ratiometric strategy to monitor the amount of NADH formation in the presence of NAD^+^, d-lactate, and the enzyme (see Materials and Methods). For TP0037, we observed a specific activity in this assay of over 5,000 units/mg/ml. Using the same assay, only about one-thousandth of the activity was observed when using l-lactate as the substrate.

**TABLE 4:** Enzymatic activity of TP0037 and its mutants

TP0037 protein	sp act (U/mg/ml)^*a*^	
	d-Lactate	l-Lactate
Wild-type	5,000 ± 200	7 ± 0
Y101A	172 ± 5	0.4 ± 0.09
R235A	NA	NA
H296A	NA	NA

a All assays were performed in triplicate; the results shown are averages ± standard deviations. NA, not available.

As a test to confirm that the putative active site residues Y101, R235, and H296 all had roles in catalysis, we individually mutated them to alanine and assessed the respective activities of the mutant proteins (Table 4). Only Y101A showed detectable activity in these assays, and its activity was about one-thirtieth that of the wild-type enzyme. All three proteins have melting temperatures similar to that of the wild-type enzyme (Table 3), ruling out gross misfolding events as the cause for their respective diminutions in enzyme activity. It therefore appears that the active site identified by structural homology (Fig. 4) is operative in TP0037.

### Bioinformatics.

**FIG 6:**
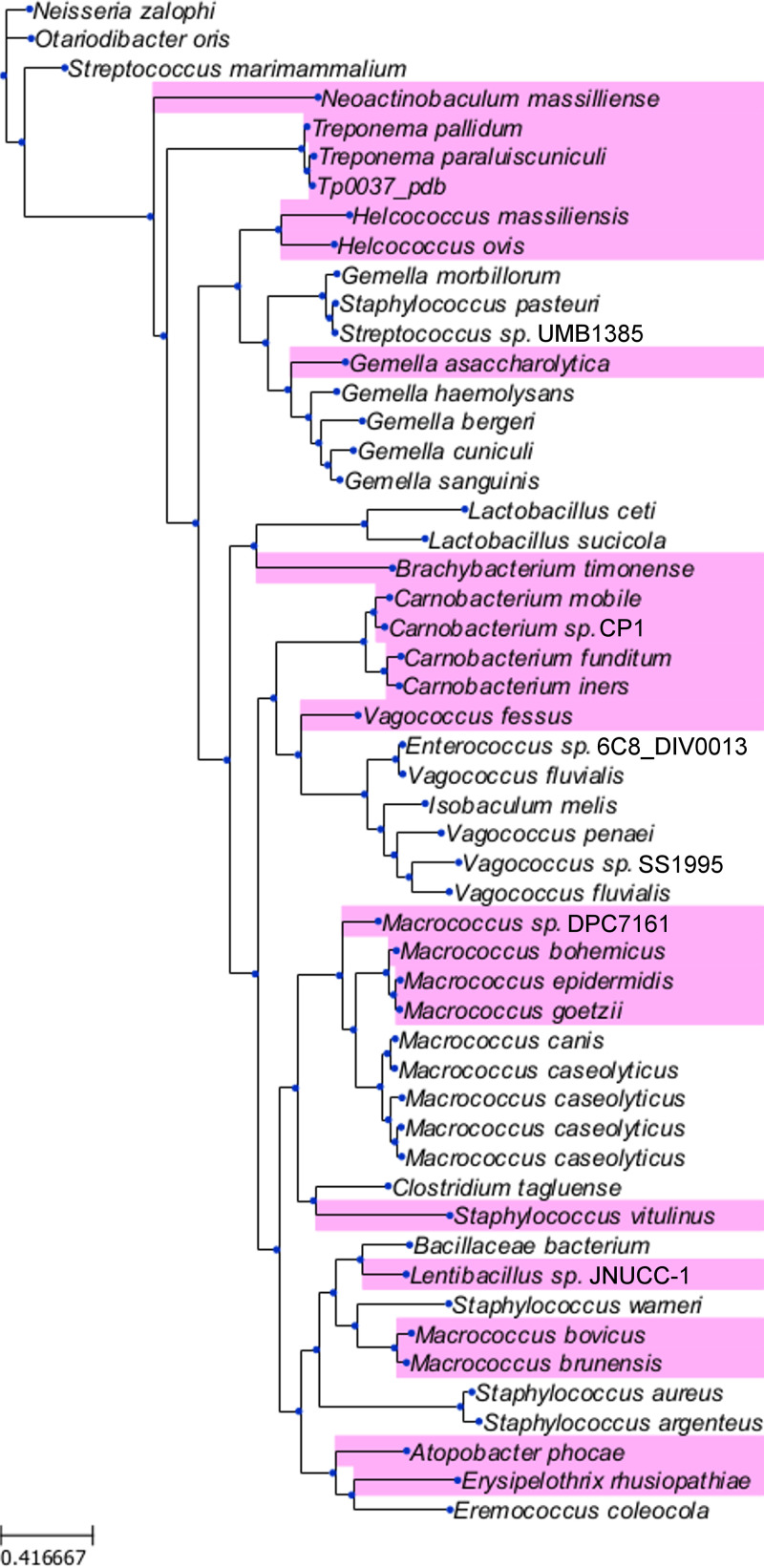
Phylogenetic tree for the most similar TP0037 homologs. The top 100 matches from a BLAST search of sequence databases using the sequence of TP0037 were culled of duplicates, aligned, and scrutinized for phylogenetic relationships as described in Materials and Methods. Organism names represent TP0037-like proteins from the respective bacteria. The organism names shown on a pink background harbored a directly adjacent (presumably co-operonic) gene encoding a TP0038-like protein. Shown at the lower left is a scale indicating the amino acid substitutions per site.

To maximize the information gleaned from the crystal structure of TP0037, we initiated a structure-based search of sequence databases for proteins most similar to the treponemal enzyme. As structurally similar d-lactate dehydrogenases are widespread in bacteria, a large number of matches from other organisms were uncovered; we examined only the top one hundred, which were ostensibly the most closely related to TP0037. From these, we constructed a molecular phylogeny (Fig. 6) to aid in discerning any patterns with respect to the sequence similarities of the TP0037-like proteins. Notably absent from these matches were d-LDHs from other treponemes; among the genus *Treponema*, only T. paraluiscuniculi and T. pallidum subspecies harbored homologs to TP0037. This finding suggested that these treponemes employ metabolic strategies that are not present in other members of the genus. Other genes for TP0037-like proteins were from a wide variety of bacteria, including free-living, commensal, and pathogenic species (Fig. 6).

Having identified these organisms as encoding TP0037 homologs, we examined the genomic neighborhood of their respective *tp0037*-like genes. Proteins from the causative agents of yaws and bejel (T. pallidum subspecies *pertenue* and *endemicum*, respectively) were not included in this analysis because the amino acid sequences of their versions of TP0037 were identical to that of T. pallidum TP0037. The *tp0037* gene appears to be co-operonic with one other gene, *tp0038*. By sequence homology, the product of the latter gene has been annotated as the enzyme IIC (EIIC) in a carbohydrate phosphotransferase system. EIICs are transmembrane proteins that transport carbohydrates across the bacterial cytoplasmic membrane and facilitate the transfer of a phosphate to them (59). This raises the intriguing prospect that TP0038 is actually a d-lactate import protein. Buttressing this notion is the fact that roughly half of other organisms flagged as carrying TP0037 homolog genes also have an adjacent gene with homology to TP0038 (Fig. 6).

## DISCUSSION

A longstanding conundrum in treponemal biology concerns how T. pallidum, a highly invasive pathogen, generates sufficient energy to fulfill its complex pathogenesis processes during human infection. For decades, it has been assumed that T. pallidum relies solely on glucose catabolism (via glycolysis) for the generation of its ATP (7, 34), a notion largely supported by genomic information (34); T. pallidum carries a gene(s) that encodes at least one d-glucose ABC-type transporter (MglB-2) (30, 60) and possesses all of the enzymes necessary for glycolysis (7, 34, 40). However, T. pallidum lacks a Krebs cycle as well as oxidative phosphorylation (34), consistent with the facts that the spirochete readily disseminates within low-oxygen (tissue) environments and is microaerophilic (61).

A hallmark feature of T. pallidum is its highly motile nature (7, 62) by virtue of its numerous bipolar flagella (endoflagella) that lie and overlap within the periplasmic space (and spin asymmetrically) (7, 42). The robust movement of the spirochete implies that motility is a key virulence factor essential for treponemal tissue invasion and host dissemination. Bacterial motility typically is dependent on abundant energy generation (63), somewhat counter to the premise that T. pallidum relies solely on glycolysis, a pathway severely limited in its ability to generate ATP from glucose. Given this line of reasoning, we have been pursuing the notion that additional energy conservation processes, either via a chemiosmotic gradient, substrate-level phosphorylation, or both, likely exist in T. pallidum to satisfy the spirochete’s presumed dynamic chemotaxis (via methyl-accepting chemotaxis proteins), motility, and tissue invasion (7, 42).

With this as a backdrop, we recently hypothesized that an answer, at least in part, to T. pallidum’s energy conundrum lies in what we have termed its “flavin-centric” metabolic lifestyle (21). Flavin-based energy conservation is garnering increased attention as a fundamental mechanism of biological energy conservation in anaerobic acetogenic bacteria (43, 64). Acetogens conventionally utilize a membrane-bound, flavin-based sodium ion-translocating ferredoxin:NAD^+^ oxidoreductase comprising a *Rhodobacter* nitrogen fixation (RNF)-type redox pump (43). The complex catalyzes electron transfer from the electron donor (reduced ferredoxin) to the electron acceptor (NAD^+^), and the negative free-energy change (the redox potential of ferredoxin is lower than that of NAD^+^) in this reaction drives the formation of a transmembrane ion gradient, which can then be used by the ATP synthase module to generate ATP (43, 65). Acetogens typically also utilize a wide variety of nutrients (including lactate, referred to herein as “lactate acetogens”) as carbon and energy sources (via substrate-level phosphorylation) and converting them to acetate (43). Many of T. pallidum’s overall proteome components are either noncanonical or cryptic, rendering them refractory to analyses by contemporary bioinformatics for the prediction of alternative energy conservation systems. In this regard, we have proposed that T. pallidum likely encodes a noncanonical RNF-type redox system (21), which is functionally akin to aerobic sodium-dependent NADH:quinone oxidoreductase (NQR) multicomponent complexes (66–69). RNF-type redox pumps rely not on quinones but on flavin to generate an electrochemical gradient across the cytoplasmic membrane (T. pallidum lacks quinone biosynthesis). The gradient generated is requisite for driving additional ATP synthesis (21, 70) (via activation of the rotary ATP synthase) as well as flagellar rotation (7, 21, 69, 71), processes both believed to be essential to T. pallidum pathogenesis.

One notable difference between classical RNF redox pumps and that proposed for T. pallidum is that because the spirochete lacks ferredoxin, the treponemal RNF complex likely relies on flavodoxin for electron transfer (21, 34, 72, 73). However, this prompts the question as to how the flavodoxin is reduced and how this reducing power is utilized by the RNF complex (Fig. 1). The answer likely is engendered by our data that now establishes T. pallidum as a d-lactate acetogen; that is, the spirochete has the ability to catabolize d-lactate to acetate. In general, d-lactate is oxidized to pyruvate by d-lactate dehydrogenase (d-LDH), followed by an oxidative decarboxylation of pyruvate to acetyl-CoA (and CO_2_) via a unique flavodoxin (Fld)-dependent pyruvate:flavodoxin oxidoreductase (PFOR) coupled with Fld reduction (note that T. pallidum lacks a conventional NAD^+^-dependent pyruvate dehydrogenase for the conversion of pyruvate to acetyl-CoA). As depicted in Fig. 1, acetyl-CoA is then likely converted to acetyl-phosphate by a putative phosphate acetyltransferase (Pta, TP0094), probably followed by the transfer of the phosphate to ADP by an acetate kinase (AckA, TP0476), resulting in the formation of ATP (via substrate-level phosphorylation) and acetate. The electron carrier (Fld) that has been reduced in this process is used for energizing the RNF module (see below).

In this scenario, unlike other acetogens, T. pallidum’s Fld-PFOR is poised strategically at the interface of its d-lactate acetogenesis and the flavin-based RNF module (where Fld is the central electron carrier). As denoted in Fig. 1, lower-midpoint potential (reduced) Fld (−450 mV) liberated by PFOR should trigger the thermodynamically favorable electron transfer to low-midpoint potential NAD^+^ (−320 mV) (74) via the flavin-based RNF complex; this negative free-energy change creates a chemiosmotic gradient across the spirochete’s cytoplasmic membrane for nonglycolytic ATP synthesis via the ATP synthase nanomachine (Fig. 1). In addition, the acetyl-phosphate intermediate of the acetogenic pathway is further metabolized to acetate for additional ATP generation via substrate-level phosphorylation (Fig. 1), thereby ensuring d-lactate-to-acetate conversion for the maintenance of cellular bioenergetics. Taken together, this coordination and cooperation of chemiosmosis and acetogenesis for metabolic adaptation not only improve overall energy generation for the pathogen but also provide strategic accommodation for T. pallidum’s metabolic lifestyle under microaerophilic tissue environments.

The first step in the conversion of d-lactate to acetate in T. pallidum is likely to be the NAD^+^-dependent oxidation of the metabolite by d-lactate dehydrogenase (d-LDH) to form pyruvate and NADH (Fig. 1). The gene product hypothesized to catalyze this reaction is TP0037. Studies detailed herein, demonstrating structural (Fig. 1 and 3, Table 2), hydrodynamic (Fig. 5), and functional (Table 4) homologies to known d-LDHs, confirm that the protein can function as a d-LDH, which is entirely consistent with the treponeme’s ability to consume d-lactate for its subsequent oxidation to pyruvate (75). We therefore propose to rename TP0037 TpD-LDH (for T. pallidum d-lactate dehydrogenase), and the current study provides strong support for the notion that this enzyme can catalyze the starting point of d-lactate acetogenesis in T. pallidum. Although lactate is a common substrate for major groups of strictly anaerobic bacteria (43, 64), its potential role in the bioenergetics of microaerophilic treponemes has remained obscure. Thus, our study clarifies the role of d-lactate utilization as an alternate carbon and energy source under changing host tissue environments (e.g., high to low O_2_), where glucose supplies could be more limited. This metabolic flexibility may play a strategic role in T. pallidum’s stealth pathogenicity.

The source of d-lactate for this pathway remains uncertain. However, bacteria in the human microbiome secrete this metabolite into the gastrointestinal tract (76), which can then be absorbed into the human bloodstream (77). d-Lactate additionally is a product of the human methylglyoxal pathway (77), which may also contribute to serum d-lactate levels. d-Lactate uptake and efflux via the monocarboxylate transporters (MCTs) can be reasonably postulated in most human (and mammalian) tissues, including the gut epithelium, skeletal muscle, liver, kidney, neuronal tissues (including the brain), retina, and various blood cells (78). Although no canonical lactate transporter is apparent in the genome of T. pallidum (34), an open reading frame for a putative carbohydrate transporter (*tp0038*; see Fig. 1) is present in the same operon as that for TpD-LDH (*tp0037*), raising the possibility that this transporter is responsible for the import of d-lactate into T. pallidum (Fig. 1). Furthermore, our gene neighborhood analysis (Fig. 6) revealed that *tp0038*-like genes are directly adjacent to *tp0037*-like genes in a diverse set of bacteria, thereby potentially establishing the existence of a treponemal-type d-lactate import/utilization system in other bacteria.

Despite the apparent broader implications of these findings across many bacterial species, T. pallidum and its subspecies stand out as the only pathogenic spirochetes to employ this metabolic/import strategy. For example, the major pathogens Treponema denticola (periodontal disease) and Borrelia burgdorferi (Lyme disease) do not possess a TP0037 homolog; instead, they appear to metabolize l-lactate, as they both have an l-lactate dehydrogenase (TDE_0351 and BB_0087, respectively). This difference may partly limit the potential use of T. denticola and B. burgdorferi as heterologous systems for the study of T. pallidum genetics and biology.

Our data in support of a flavin-dependent d-lactate acetogenic energy conservation pathway is a key feature at the core of T. pallidum’s flavin-centric metabolic lifestyle, in which d-lactate is implicated as a previously unrecognized energy source. This addresses, at least in part, the decades-long energetics enigma imposed by the microaerophilicity of T. pallidum, which does not possess cytochromes, quinones, or ferredoxin as electron carriers. Therefore, the role of flavin as a central electron carrier in the d-lactate acetogenic energy conservation pathway (Fig. 1) further validates our proposed flavin-centric metabolic lifestyle that subserves T. pallidum’s stealth pathogenicity. The other treponemal enzymes involved in the acetogenic energy conservation pathway would be pyruvate:flavodoxin oxidoreductase (PFOR), phosphate acetyltransferase (Pta), and acetate kinase (AckA). The genes putatively encoding PFOR, Pta, and AckA are *tp0939*, *tp0094*, and *tp0476*, respectively. Future structural, biophysical, and biochemical experiments on the products of these genes are warranted to confirm these respective functions. Our current studies also have laid a foundation for potentially exploiting the recently described long-term *in vitro* culture system for T. pallidum (16) for further substantiating the roles of selected enzymes in key treponemal metabolic processes.

## MATERIALS AND METHODS

### Cloning, protein expression, and protein purification.

To produce a recombinant derivative of TP0037 in Escherichia coli, the DNA fragment encoding amino acid resides 1 to 331 of TP0037 was PCR amplified from Treponema pallidum genomic DNA, the fragment was digested with BamHI and ligated into BamHI-cut expression vector pProEX-HTb. E. coli BL21(DE3) competent cells were transformed with the ligated DNA products and selected for ampicillin resistance on LB agar plates. Cloning junctions/fragments were verified by DNA sequencing. E. coli BL21(DE3) cells were grown at 37°C in LB medium containing 100 μg/ml of ampicillin until the cell density reached an *A*_600_ of ∼0.6. The cells were then induced for ∼20 h with 0.6 mM isopropyl-β-d-thiogalactopyranoside (IPTG) at 16°C and harvested, and the cell pellets were stored at −80°C. The procedures for expression and purification of the recombinant proteins were essentially as previously described (22, 29, 37). The final storage buffer for the protein comprised 20 mM HEPES (pH 7.5), 100 mM NaCl, and 2 mM *n*-octyl-β-d-glucopyranoside (BOG).

For the construction of structure-guided recombinant TP0037 (rTP0037) variants, the R235A, Y101A, and H296A mutations were individually introduced into the plasmid carrying the wild-type *tp0037* sequence using the QuikChange site-directed mutagenesis kit (Agilent Technologies). The mutation was confirmed by DNA sequencing. The mutant protein was expressed and purified as described above. Protein concentrations were determined in storage buffer using UV absorption at 280 nm. Extinction coefficients were calculated from the protein sequences using the ProtParam tool of ExPASy server (www.expasy.org).

### Crystallization, data collection, and structure determination.

Using the sitting-drop vapor-diffusion technique in 96-well plates with Structure 1 and 2 (Molecular Dimensions), JCSG plus (Qiagen), PACT (Molecular Dimensions), and Index (Hampton Research) crystallization suites and a crystallization robot (Crystal Gryphon), the initial crystallization conditions were determined at 20°C. Crystallization screens for TP0037 with a protein concentration of ∼20 mg/ml (in buffer A) yielded diffraction-quality crystals in C7 well of Structure 1 and 2 suite (200 mM sodium-potassium phosphate, 100 mM *N*,*N*-methylenebisacrylamide [BIS]-Tris propane [pH 7.5], 20% [wt/vol] polyethylene glycol 3350 [PEG 3350]) after 7 days. The crystals were transferred to the stabilization buffer containing 200 mM sodium-potassium tartrate, 100 mM BIS-Tris propane (pH 7.5), 100 mM NaCl, 2 mM BOG, 20% PEG 3350, and various concentrations of ethylene glycol. For cryoprotection, crystals were serially transferred to solutions containing 5%, 10%, and 15% (vol/vol) ethylene glycol. After about 1 min in this final solution, the crystals were flash-cooled in liquid nitrogen and stored until data collection.

Diffraction data with a *d*_min_ spacing of 1.38 Å were acquired from these crystals at beamline 19-ID at the Structural Biology Center of the Advanced Photon Source at Argonne National Laboratories (Table 1). These data revealed that the crystals had the symmetry of space group P2_1_2_1_2_1_. The data were indexed, integrated, and scaled using HKL2000 (79), followed by a treatment to put them on an absolute scale and eliminate negative intensities (80, 81). To locate the best search model, a hidden-Markov-based search of sequence databases was performed with the TP0037 amino acid sequence; the top hit, the (*R*)-2-hydroxyglutarate dehydrogenase from Acidaminococcus fermentans (PDB accession code 1XDW [82, 83]) was used after removing hetero-atoms and trimming side chains that were not identical to those in TP0037 down to the last common atom. The PHENIX GUI (84) implementation of Phaser (85) was used to calculate phases using molecular replacement, resulting in a final log likelihood gain of 251 and a translation function Z-score of 14.9. The quality of the initial difference electron density map was poor, but the model could be completely built using the web-based ARP/wARP server (86). Subsequently, the model was refined in PHENIX using the rigid-body refinement, simulated annealing, positional refinement, and anisotropic atomic displacement parameter (ADP) refinement protocols. Riding hydrogen atoms were used at all stages of refinement, and weights for the geometric and ADP terms in the maximum likelihood algorithms were refined. Coot (87) was used to adjust the model between rounds of refinement. MolProbity (88) was used for structure validation. The final model contains 661 amino acids (i.e., the dimer of TP0037), 6 molecules of ethylene glycol, 2 chloride ions, and 643 water molecules. All structure-containing figures were rendered using PyMol (Schrödinger, LLC).

### Dynamic and static light scattering.

All DLS and SLS experiments were carried out in a Dynapro Nanostar instrument (Wyatt Technologies) at 25°C in storage buffer. The sample (5 μl at 1.54 mg/ml) was dispensed into a quartz cuvette, and the scattered light intensity and fluctuations thereof were simultaneously monitored. The results reported were from three technical replicates on the same sample. SLS data were analyzed in Dynamics version 7.5.0.17 (Wyatt Technologies) using a *dn*/*dc* value of 0.1887 ml/g. The DLS data were analyzed using Dynamics, which was used in the regularization mode to calculate hydrodynamic radius distributions as shown in Fig. 5A.

### Analytical ultracentrifugation.

All analytical ultracentrifugation (AUC) studies were conducted in a Beckman-Coulter XL-I ultracentrifuge at 20°C. Volumes of 400 μl of various concentrations of TP0037 in storage buffer were loaded into the sample sectors of charcoal-filled Epon centerpieces that were positioned between sapphire windows in standard aluminum housings. The same volume of storage buffer without protein was applied to the sample sectors. The assembled and sealed AUC cells were inserted into an An50-Ti rotor, which was then placed into the centrifuge. The samples were allowed to equilibrate at the experimental temperature for 2.5 h prior to centrifugation. The experiment was commenced by accelerating the rotor to 50,000 rpm; after the target rotor speed had been achieved, data acquisition was initiated utilizing the absorbance optics tuned to 280 nm. Data were collected overnight. The data were analyzed using the *c*(*s*) model available in SEDFIT (56, 57), with a regularization level of 0.683 and a resolution of 150.

### Differential scanning fluorimetry.

To assess protein stability in the presence of ligands, differential scanning fluorimetry (DSF) of TP0037 and its variants were performed in a 96-well PCR plate (Bio-Rad) using a real-time PCR instrument (Bio-Rad). For binding assays, ligands were prepared freshly in sterile water. Each 25-μl standard assay mixture in a 96-well PCR plate contained 20 μl purified TP0037 and SYPRO Orange (5,000× stock solution; Life Technologies) at 5× concentration in a storage buffer. Five microliters of ligands were added in each well to final concentrations of 5 mM NAD and 20 mM either d-lactate or l-lactate. Samples were heat denatured from 4°C to 85°C in 0.5°C steps. The protein unfolding curves were monitored from the differential fluorescence changes (−Δ*F*) of protein-bound SYPRO Orange. The first derivative values (−Δ*F*/Δ*T*) reported by the instrument’s software were used to find the minimum value. The derivative data from this temperature, along with the preceding and succeeding five data points were fitted with a second-degree polynomial, and this fitting equation was analyzed to find the true minimum, i.e., the point at which the first derivative of the polynomial equation was 0, which was taken as the *T_m_*. All such experiments were performed in triplicate, and the reported *T_m_* values were the means of the replicates.

### Enzyme assays.

Lactate dehydrogenase activity was assayed using commercially available kits (Amplite colorimetric d-lactate dehydrogenase assay kit and Amplite colorimetric l-lactate dehydrogenase assay kit; AAT Bioquest) according to the manufacturer’s instructions. Briefly, for the d-lactate assays, the provided NAD was suspended in 100 μl to form a 200× solution. All reactions were carried out in the wells of 96-well plates; each well contained 100 μl, composed of 0.5 μl NAD, a volume needed to supply the desired protein mass (10 ng, 1 μg, 10 μg, and 10 μg for wild-type, Y101A, R235A, and H236A, respectively), with the remainder of the volume comprising the manufacturer’s supplied assay buffer. For the l-lactate assays, an identical procedure was followed, except that 1 μg of the subject enzyme was added. The assays were incubated at 37°C for 30 min, followed by detection by monitoring increase in the *A*_575_/*A*_605_ ratio in a SPECTRAmax Plus plate reader (Molecular Devices). This value was compared to a standard curve (all readings were in the linear range of the standard curve) to arrive at the number of units of activity, which we normalized by the final concentration of the protein in the assay.

### Bioinformatics.

Protein sequence databases were searched using BLAST (89). The top 100 hits from this search were further culled, eliminating redundant sequences. The surviving sequences were aligned using a structure-based alignment technique (PROMALS3D) (90), and the sequence of TP0037 was represented twice in the alignment, once from the BLAST search and once from the sequence taken from the PDB file used for the structure-based alignment. After conversion of the result to Phylip format (insilico.ehu.es/tophylip/), PhyML was used to construct the cladogram, using the LG model for amino acid substitution (91). The cladogram was visualized and rendered with the Python library ETE version 3.1.1 (92).

### Data availability.

The crystal structure reported herein has been deposited in the Protein Data Bank with accession number 7JP2 (www.rcsb.org/structure/7JP2 [93]). Custom Python code (Python 2.7) for determining *T_m_* from DSF data is available upon request.

## References

[1] Simms I, Fenton KA, Ashton M, Turner KME, Crawley-Boevey EE, Gorton R, Thomas DR, Lynch A, Winter A, Fisher MJ, Lighton L, Maguire HC, Solomou M 2005 The re-emergence of syphilis in the United Kingdom: the new epidemic phases. Sex Transm Dis 32:220–226. doi:10.1097/01.olq.0000149848.03733.c1. 15788919

[2] Bremer V, Marcus U, Hamouda O 2012 Syphilis on the rise again in Germany − results from surveillance data for 2011. Euro Surveill 17(29):20222 https://www.eurosurveillance.org/content/10.2807/ese.17.29.20222-en. 22835467

[3] Velicko I, Unemo M 2012 Recent trends in gonorrhoea and syphilis epidemiology in Sweden: 2007 to 2011. Euro Surveill 17(29):20223 https://www.eurosurveillance.org/content/10.2807/ese.17.29.20223-en. 22835468

[4] Cohen MS, Hawkes S, Mabey D 2006 Syphilis returns to China…with a vengeance. Sex Transm Dis 33:724–725. doi:10.1097/01.olq.0000245917.47692.b7. 17130806

[5] World Health Organization. 2011 Prevalence and incidence of selected sexually transmitted infections: *Chlamydia trachomatis*, *Neisseria gonorrhoeae*, syphilis and *Trichomonas vaginalis*. Methods and results used by WHO to generate 2005 estimates. World Health Organization, Geneva, Switzerland.

[6] World Health Organization. 2016 WHO guidelines for the treatment of *Treponema pallidum* (syphilis). World Health Organization, Geneva, Switzerland. 27631046

[7] Radolf JD, Deka RK, Anand A, Smajs D, Norgard MV, Yang XF 2016 *Treponema pallidum*, the syphilis spirochete: making a living as a stealth pathogen. Nat Rev Microbiol 14:744–759. doi:10.1038/nrmicro.2016.141. 27721440 PMC5106329

[8] Bowen V, Su J, Torrone E, Kidd S, Weinstock H 2015 Increase in incidence of congenital syphilis – United States, 2012–2014. MMWR Morb Mortal Wkly Rep 64:1241–1245. doi:10.15585/mmwr.mm6444a3. 26562206

[9] Kerani RP, Handsfield HH, Stenger MS, Shafii T, Zick E, Brewer D, Golden MR 2007 Rising rates of syphilis in the era of syphilis elimination. Sex Transm Dis 34:154–161. doi:10.1097/01.olq.0000233709.93891.e5. 17179773

[10] Centers for Disease Control and Prevention. 2017 Sexually transmitted disease surveillance 2016. Centers for Disease Control and Prevention, Atlanta, GA.

[11] Centers for Disease Control and Prevention. 2009 Primary and secondary syphilis – Jefferson County, Alabama, 2002–2007. Morb Mortal Wkly Rep 58:463–467. 19444149

[12] Peeling RW, Mabey D, Kamb ML, Chen XS, Radolf JD, Benzaken AS 2017 Syphilis. Nat Rev Dis Primers 3:17073. doi:10.1038/nrdp.2017.73. 29022569 PMC5809176

[13] Henkel JS, Davis J, Farley N 2020 Anatomical and biochemical evidence for *Treponema pallidum* in a 19th to early twentieth century skeletal cadaver. Forensic Sci Med Pathol doi:10.1007/s12024-020-00243-2. 32394208

[14] Tampa M, Sarbu I, Matei C, Benea V, Georgescu SR 2014 Brief history of syphilis. J Med Life 7:4–10. PMC3956094 24653750

[15] Norris SJ 1993 Polypeptides of *Treponema pallidum*: progress toward understanding their structural, functional, and immunologic roles. Microbiol Rev 57:750–779. doi:10.1128/MMBR.57.3.750-779.1993. 8246847 PMC372934

[16] Edmondson DG, Hu B, Norris SJ 2018 Long-term *in vitro* culture of the syphilis spirochete *Treponema pallidum* subsp. *pallidum*. mBio 9:e01153-18. doi:10.1128/mBio.01153-18. 29946052 PMC6020297

[17] Deka RK, Neil L, Hagman KE, Machius M, Tomchick DR, Brautigam CA, Norgard MV 2004 Structural evidence that the 32-kilodalton lipoprotein (Tp32) of *Treponema pallidum* is an L-methionine-binding protein. J Biol Chem 279:55644–55650. doi:10.1074/jbc.M409263200. 15489229

[18] Brautigam CA, Deka RK, Liu WZ, Tomchick DR, Norgard MV 2017 Functional clues from the crystal structure of an orphan periplasmic ligand-binding protein from *Treponema pallidum*. Protein Sci 26:847–856. doi:10.1002/pro.3133. 28168761 PMC5368063

[19] Brautigam CA, Deka RK, Liu WZ, Norgard MV 2015 Insights into the potential function and membrane organization of the TP0435 (Tp17) lipoprotein from *Treponema pallidum* derived from structural and biophysical analyses. Protein Sci 24:11–19. doi:10.1002/pro.2576. 25287511 PMC4282407

[20] Lee Y-H, Deka RK, Norgard MV, Radolf JD, Hasemann CA 1999 *Treponema pallidum* TroA is a periplasmic zinc-binding protein with a helical backbone. Nat Struct Biol 184:628–633. 10.1038/10677 10404217

[21] Deka RK, Brautigam CA, Liu WZ, Tomchick DR, Norgard MV 2015 Evidence for posttranslational protein flavinylation in the syphilis spirochete *Treponema pallidum*: structural and biochemical insights from the catalytic core of a periplasmic flavin-trafficking protein. mBio 6:e00519-15. doi:10.1128/mBio.00519-15. 25944861 PMC4436053

[22] Deka RK, Brautigam CA, Goldberg M, Schuck P, Tomchick DR, Norgard MV 2012 Structural, bioinformatic, and *in vivo* analyses of two *Treponema pallidum* lipoproteins reveal a unique TRAP transporter. J Mol Biol 416:678–696. doi:10.1016/j.jmb.2012.01.015. 22306465 PMC3289903

[23] Brautigam CA, Deka RK, Schuck P, Tomchick DR, Norgard MV 2012 Structural and thermodynamic characterization of the interaction between two periplasmic *Treponema pallidum* lipoproteins that are components of a TPR-protein-associated TRAP transporter (TPAT). J Mol Biol 420:70–86. doi:10.1016/j.jmb.2012.04.001. 22504226 PMC3367087

[24] Deka RK, Machius M, Norgard MV, Tomchick DR 2002 Crystal structure of the 47-kDa lipoprotein of *Treponema pallidum* reveals a novel penicillin-binding protein. J Biol Chem 277:41857–41864. doi:10.1074/jbc.M207402200. 12196546

[25] Deka RK, Brautigam CA, Yang XF, Blevins JS, Machius M, Tomchick DR, Norgard MV 2006 The PnrA (Tp0319; TmpC) lipoprotein represents a new family of bacterial purine nucleoside receptor encoded within an ATP-binding cassette (ABC)-like operon in *Treponema pallidum*. J Biol Chem 281:8072–8081. doi:10.1074/jbc.M511405200. 16418175

[26] Machius M, Brautigam CA, Tomchick DR, Ward P, Otwinowski Z, Blevins JS, Deka RK, Norgard MV 2007 Structural and biochemical basis for polyamine binding to the Tp0655 lipoprotein of *Treponema pallidum*: putative role for Tp0655 (TpPotD) as a polyamine receptor. J Mol Biol 373:681–694. doi:10.1016/j.jmb.2007.08.018. 17868688 PMC2094014

[27] Deka RK, Brautigam CA, Biddy BA, Liu WZ, Norgard MV 2013 Evidence for an ABC-type riboflavin transporter system in pathogenic spirochetes. mBio 4:e00615-12. doi:10.1128/mBio.00615-12. 23404400 PMC3573665

[28] Deka RK, Brautigam CA, Liu WZ, Tomchick DR, Norgard MV 2013 The TP0796 lipoprotein of *Treponema pallidum* is a bimetal-dependent FAD pyrophosphatase with a potential role in flavin homeostasis. J Biol Chem 288:11106–11121. doi:10.1074/jbc.M113.449975. 23447540 PMC3630870

[29] Deka RK, Brautigam CA, Liu WZ, Tomchick DR, Norgard MV 2016 Molecular insights into the enzymatic diversity of flavin-trafficking protein (Ftp; formerly ApbE) in flavoprotein biogenesis in the bacterial periplasm. Microbiologyopen 5:21–38. doi:10.1002/mbo3.306. 26626129 PMC4767422

[30] Brautigam CA, Deka RK, Liu WZ, Norgard MV 2016 The Tp0684 (MglB-2) lipoprotein of *Treponema pallidum*: a glucose-binding protein with divergent topology. PLoS One 11:e0161022. doi:10.1371/journal.pone.0161022. 27536942 PMC4990184

[31] Brautigam CA, Deka RK, Liu WZ, Norgard MV 2018 Crystal structures of MglB-2 (TP0684), a topologically variant D-glucose-binding protein from *Treponema pallidum*, reveal a ligand-induced conformational change. Protein Sci 27:880–885. doi:10.1002/pro.3373. 29318719 PMC5866939

[32] Haake DA, Zückert WR 2017 Spirochetal lipoproteins in pathogenesis and immunity. Curr Top Microbiol Immunol 415:239–271. doi:10.1007/82_2017_78. 29196824

[33] Kovacs-Simon A, Titball RW, Michell SL 2011 Lipoproteins of bacterial pathogens. Infect Immun 79:548–561. doi:10.1128/IAI.00682-10. 20974828 PMC3028857

[34] Fraser CM, Norris SJ, Weinstock GM, White O, Sutton GG, Dodson R, Gwinn M, Hickey EK, Clayton R, Ketchum KA, Sodergren E, Hardham JM, McLeod MP, Salzberg S, Peterson J, Khalak H, Richardson D, Howell JK, Chidambaram M, Utterback T, McDonald L, Artiach P, Bowman C, Cotton MD, Fujii C, Garland S, Hatch B, Horst K, Roberts K, Sandusky M, Weidman J, Smith HO, Venter JC 1998 Complete genome sequence of *Treponema pallidum*, the syphilis spirochete. Science 281:375–388. doi:10.1126/science.281.5375.375. 9665876

[35] Setubal JC, Reis M, Matsunaga J, Haake DA 2006 Lipoprotein computational prediction in spirochaetal genomes. Microbiology 152:113–121. doi:10.1099/mic.0.28317-0. 16385121 PMC2667199

[36] Brautigam CA, Deka RK, Ouyang Z, Machius M, Knutsen G, Tomchick DR, Norgard MV 2012 Biophysical and bioinformatic analyses implicate the *Treponema pallidum* Tp34 lipoprotein (Tp0971) in transition metal homeostasis. J Bacteriol 194:6771–6781. doi:10.1128/JB.01494-12. 23042995 PMC3510569

[37] Deka RK, Brautigam CA, Tomson FL, Lumpkins SB, Tomchick DR, Machius M, Norgard MV 2007 Crystal structure of the Tp34 (TP0971) lipoprotein of *Treponema pallidum*: implications of its metal-bound state and affinity for human lactoferrin. J Biol Chem 282:5944–5958. doi:10.1074/jbc.M610215200. 17192261

[38] Bogachev AV, Baykov AA, Bertsova YV 2018 Flavin transferase: the maturation factor of flavin-containing oxidoreductases. Biochem Soc Trans 46:1161–1169. doi:10.1042/BST20180524. 30154099

[39] Åstrand M, Cuellar J, Hytönen J, Salminen TA 2019 Predicting the ligand-binding properties of *Borrelia burgdorferi* s.s. Bmp proteins in light of the conserved features of related *Borrelia* proteins. J Theor Biol 462:97–108. doi:10.1016/j.jtbi.2018.11.004. 30419249

[40] Norris SJ, Cox DL, Weinstock GM 2001 Biology of *Treponema pallidum*: correlation of functional activities with genome sequence data. J Mol Microbiol Biotechnol 3:37–62. 11200228

[41] Liu J, Howell JK, Bradley SD, Zheng Y, Zhou ZH, Norris SJ 2010 Cellular architecture of *Treponema pallidum*: novel flagellum, periplasmic cone, and cell envelope as revealed by cryo electron tomography. J Mol Biol 403:546–561. doi:10.1016/j.jmb.2010.09.020. 20850455 PMC2957517

[42] LaFond RE, Lukehart SA 2006 Biological basis for syphilis. Clin Microbiol Rev 19:29–49. doi:10.1128/CMR.19.1.29-49.2006. 16418521 PMC1360276

[43] Schuchmann K, Müller V 2016 Energetics and application of heterotrophy in acetogenic bacteria. Appl Environ Microbiol 82:4056–4069. doi:10.1128/AEM.00882-16. 27208103 PMC4959221

[44] Stoll VS, Kimber MS, Pai EF 1996 Insights into substrate binding by D-2-ketoacid dehydrogenases from the structure of *Lactobacillus pentosus* D-lactate dehydrogenase. Structure 4:437–447. doi:10.1016/s0969-2126(96)00049-4. 8740366

[45] Razeto A, Kochhar S, Hottinger H, Dauter M, Wilson KS, Lamzin VS 2002 Domain closure, substrate specificity and catalysis of D-lactate dehydrogenase from *Lactobacillus bulgaricus*. J Mol Biol 318:109–119. doi:10.1016/S0022-2836(02)00086-4. 12054772

[46] Krissinel E, Henrick K 2007 Inference of macromolecular assemblies from crystalline state. J Mol Biol 372:774–797. doi:10.1016/j.jmb.2007.05.022. 17681537

[47] Krissinel E, Henrick K 2004 Secondary-structure matching (SSM), a new tool for fast protein structure alignment in three dimensions. Acta Crystallogr D Biol Crystallogr 60:2256–2268. doi:10.1107/S0907444904026460. 15572779

[48] Holm L, Rosenström P 2010 Dali server: conservation mapping in 3D. Nucleic Acids Res 38:W545–W549. doi:10.1093/nar/gkq366. 20457744 PMC2896194

[49] Bo Y, Hui D, Xiang L 2015 A novel D-lactate dehydrogenase from Sporolactobacillus sp. RCSB PDB doi:10.2210/pdb4xkj/pdb. (accession no. 4XKJ).

[50] Razeto A, Kochhar S, Hottinger H, Dauter M, Wilson KS, Lamzin VS 2002 Insights into domain closure, substrate specificity and catalysis of D-lactate dehydrogenase from Lactobacillus bulgaricus. RCSB PDB doi:10.2210/pdb1J4A/pdb. (accession no. 1J4A). 12054772

[51] Razeto A, Kochhar S, Hottinger H, Dauter M, Wilson KS, Lamzin VS 2002 Insights into domain closure, substrate specificity and catalysis of D-lactate dehydrogenase from Lactobacillus bulgaricus. RCSB PDB doi:10.2210/pdb1J49/pdb. (accession no. 1J49). 12054772

[52] Kochhar S, Chuard N, Hottinger H 1992 Cloning and overexpression of the *Lactobacillus bulgaricus* NAD^+^-dependent D-lactate dehydrogenase gene in Escherichia coli: purification and characterization of the recombinant enzyme. Biochem Biophys Res Commun 185:705–712. doi:10.1016/0006-291x(92)91683-h. 1610363

[53] Le Bras G, Garel JR 1991 Properties of D-lactate dehydrogenase from *Lactobacillus bulgaricus*: a possible different evolutionary origin for the D- and L-lactate dehydrogenases. FEMS Microbiol Lett 79:89–93. doi:10.1016/0378-1097(91)90533-G. 2044942

[54] Kochhar S, Hunziker PE, Leong-Morgenthaler P, Hottinger H 1992 Primary structure, physicochemical properties, and chemical modification of NAD^+^-dependent D-lactate dehydrogenase. J Biol Chem 267:8499–8513. 1569100

[55] Zhu L, Xu X, Wang L, Dong H, Yu B, Ma Y 2015 NADP^+^-preferring D-lactate dehydrogenase from *Sporolactobacillus inulinus*. Appl Environ Microbiol 81:6294–6301. doi:10.1128/AEM.01871-15. 26150461 PMC4542229

[56] Schuck P 2000 Size distribution analysis of macromolecules by sedimentation velocity ultracentrifugation and Lamm equation modeling. Biophys J 78:1606–1619. doi:10.1016/S0006-3495(00)76713-0. 10692345 PMC1300758

[57] Schuck P, Perugini MA, Gonzales NR, Howlett GJ, Schubert D 2002 Size-distribution analysis of proteins by analytical ultracentrifugation: strategies and application to model systems. Biophys J 82:1096–1111. doi:10.1016/S0006-3495(02)75469-6. 11806949 PMC1301916

[58] Niesen FH, Berglund H, Vedadi M 2007 The use of differential scanning fluorimetry to detect ligand interactions that promote protein stability. Nat Protoc 2:2212–2221. doi:10.1038/nprot.2007.321. 17853878

[59] Saier MH, Reizer J 1992 Proposed uniform nomenclature for the proteins and protein domains of the bacterial phosphoenolpyruvate: sugar phosphotransferase system. J Bacteriol 174:1433–1438. doi:10.1128/jb.174.5.1433-1438.1992. 1537788 PMC206537

[60] Deka RK, Goldberg MS, Hagman KE, Norgard MV 2004 The Tp38 (TpMg1B-2) lipoprotein binds glucose in a manner consistent with receptor function in *Treponema pallidum*. J Bacteriol 186:2303–2308. doi:10.1128/jb.186.8.2303-2308.2004. 15060032 PMC412163

[61] Cover WH, Norris SJ, Miller JN 1982 The microaerophilic nature of *Treponema pallidum*: enhanced survival and incorporation of tritiated adenine under microaerobic conditions in the presence or absence of reducing compounds. Sex Transm Dis 9:1–8. doi:10.1097/00007435-198201000-00001. 10328016

[62] Canale-Parola E 1978 Motility and chemotaxis of spirochetes. Annu Rev Microbiol 32:69–99. doi:10.1146/annurev.mi.32.100178.000441. 360979

[63] Mitchell JG, Kogure K 2006 Bacterial motility: links to the environment and a driving force for microbial physics. FEMS Microbiol Ecol 55:3–16. doi:10.1111/j.1574-6941.2005.00003.x. 16420610

[64] Weghoff MC, Bertsch J, Müller V 2015 A novel mode of lactate metabolism in strictly anaerobic bacteria. Environ Microbiol 17:670–677. doi:10.1111/1462-2920.12493. 24762045

[65] Schoelmerich MC, Katsyv A, Dönig J, Hackmann TJ, Müller V 2020 Energy conservation involving 2 respiratory circuits. Proc Natl Acad Sci U S A 117:1167–1173. doi:10.1073/pnas.1914939117. 31879356 PMC6969491

[66] Barquera B 2014 The sodium pumping NADH:quinone oxidoreductase (Na+-NQR), a unique redox-driven ion pump. J Bioenerg Biomembr 46:289–298. doi:10.1007/s10863-014-9565-9. 25052842

[67] Verkhovsky MI, Bogachev AV 2010 Sodium-translocating NADH:quinone oxidoreductase as a redox-driven ion pump. Biochim Biophys Acta 1797:738–746. doi:10.1016/j.bbabio.2009.12.020. 20056102

[68] Steuber J, Vohl G, Casutt MS, Vorburger T, Diederichs K, Fritz G 2014 Structure of the V. cholerae Na^+^-pumping NADH:quinone oxidoreductase. Nature 516:62–67. doi:10.1038/nature14003. 25471880

[69] Reyes-Prieto A, Barquera B, Juárez O 2014 Origin and evolution of the sodium-pumping NADH: ubiquinone oxidoreductase. PLoS One 9:e96696. doi:10.1371/journal.pone.0096696. 24809444 PMC4014512

[70] Mayer F, Müller V 2014 Adaptations of anaerobic archaea to life under extreme energy limitation. FEMS Microbiol Rev 38:449–472. doi:10.1111/1574-6976.12043. 24118021

[71] Häse CC, Fedorova ND, Galperin MY, Dibrov PA 2001 Sodium ion cycle in bacterial pathogens: evidence from cross-genome comparisons. Microbiol Mol Biol Rev 65:353–370. doi:10.1128/MMBR.65.3.353-370.2001. 11528000 PMC99031

[72] Buckel W, Thauer RK 2018 Flavin-based electron bifurcation, ferredoxin, flavodoxin, and anaerobic respiration with protons (Ech) or NAD^+^(Rnf) as electron acceptors: a historical review. Front Microbiol 9:401. doi:10.3389/fmicb.2018.00401. 29593673 PMC5861303

[73] Chowdhury NP, Klomann K, Seubert A, Buckel W 2016 Reduction of flavodoxin by electron bifurcation and sodium ion-dependent reoxidation by NAD^+^ catalyzed by ferredoxin-NAD^+^ reductase (Rnf). J Biol Chem 291:11993–12002. doi:10.1074/jbc.M116.726299. 27048649 PMC4933252

[74] Pueyo JJ, Gomez-Moreno C, Mayhew SG 1991 Oxidation‐reduction potentials of ferredoxin‐NADP^+^ reductase and flavodoxin from *Anabaena* PCC 7119 and their electrostatic and covalent complexes. Eur J Biochem 202:1065–1071. doi:10.1111/j.1432-1033.1991.tb16471.x. 1765067

[75] Austin FE, Cox CD 1986 Lactate oxidation by *Treponema pallidum*. Curr Microbiol 13:123–128. doi:10.1007/BF01568506.

[76] Halperin ML, Kamel KS 1996 D-lactic acidosis: turning sugar into acids in the gastrointestinal tract. Kidney Int 49:1–8. doi:10.1038/ki.1996.1. 8770942

[77] Ewaschuk JB, Naylor JM, Zello GA 2005 D-lactate in human and ruminant metabolism. J Nutr 135:1619–1625. doi:10.1093/jn/135.7.1619. 15987839

[78] Halestrap AP, Wilson MC 2012 The monocarboxylate transporter family — role and regulation. IUBMB Life 64:109–119. doi:10.1002/iub.572. 22162139

[79] Otwinowski Z, Minor W 1997 Processing of X-ray diffraction data collected in oscillation mode. Methods Enzymol 276:307–326. doi:10.1016/S0076-6879(97)76066-X. 27754618

[80] French S, Wilson K 1978 On the treatment of negative intensity observations. Acta Crystallogr A 34:517–525. doi:10.1107/S0567739478001114.

[81] Collaborative Computational Project Number 4. 1994 The CCP4 suite: programs for protein crystallography. Acta Crystallogr D Biol Crystallogr 50:760–763. doi:10.1107/S0907444994003112. 15299374

[82] Martins BM, Macedo-Ribeiro S, Bresser J, Buckel W, Messerschmidt A 2005 Structural basis for stereo-specific catalysis in NAD^+^-dependent (R)-2-hydroxyglutarate dehydrogenase from *Acidaminococcus fermentans*. FEBS J 272:269–281. doi:10.1111/j.1432-1033.2004.04417.x. 15634349

[83] Martins BM, Macedo-Ribeiro S, Bresser J, Buckel W, Messerschmidt A 2005 NAD+-dependent (R)-2-hydroxyglutarate dehydrogenase from *Acidaminococcus fermentans*. RCSB PDB doi:10.2210/pdb1XDW/pdb. (accession no. 1XDW). 15634349

[84] Adams PD, Afonine PV, Bunkóczi G, Chen VB, Davis IW, Echols N, Headd JJ, Hung W, Kapral GJ, Grosse-Kunstleve RW, McCoy AJ, Moriarty NW, Oeffner R, Read RJ, Richardson DC, Richardson JS, Terwilliger TC, Zwart PH 2010 PHENIX: a comprehensive Python-based system for macromolecular structure determination. Acta Crystallogr D Biol Crystallogr 66:213–221. doi:10.1107/S0907444909052925. 20124702 PMC2815670

[85] McCoy AJ, Grosse-Kunstleve RW, Adams PD, Winn MD, Storoni LC, Read RJ 2007 Phaser crystallographic software. J Appl Crystallogr 40:658–674. doi:10.1107/S0021889807021206. 19461840 PMC2483472

[86] Langer G, Cohen SX, Lamzin VS, Perrakis A 2008 Automated macromolecular model building for X-ray crystallography using ARP/wARP version 7. Nat Protoc 3:1171–1179. doi:10.1038/nprot.2008.91. 18600222 PMC2582149

[87] Emsley P, Cowtan K 2004 Coot: model-building tools for molecular graphics. Acta Crystallogr D Biol Crystallogr 60:2126–2132. doi:10.1107/S0907444904019158. 15572765

[88] Chen VB, Arendall WB III, Headd JJ, Keedy DA, Immormino RM, Kapral GJ, Murray LW, Richardson JS, Richardson DC 2010 MolProbity: all-atom structure validation for macromolecular crystallography. Acta Crystallogr D Biol Crystallogr 66:12–21. doi:10.1107/S0907444909042073. 20057044 PMC2803126

[89] Altschul SF, Madden TL, Schäffer AA, Zhang J, Zhang Z, Miller W, Lipman DJ 1997 Gapped BLAST and PSI-BLAST: a new generation of protein database search programs. Nucleic Acids Res 25:3389–3402. doi:10.1093/nar/25.17.3389. 9254694 PMC146917

[90] Pei J, Kim B-H, Grishin NV 2008 PROMALS3D: a tool for multiple sequence and structure alignment. Nucleic Acids Res 36:2295–2300. doi:10.1093/nar/gkn072. 18287115 PMC2367709

[91] Guindon S, Dufayard JF, Lefort V, Anisimova M, Hordijk W, Gascuel O 2010 New algorithms and methods to estimate maximum-likelihood phylogenies: assessing the performance of PhyML 3.0. Syst Biol 59:307–321. doi:10.1093/sysbio/syq010. 20525638

[92] Huerta-Cepas J, Serra F, Bork P 2016 ETE 3: reconstruction, analysis and visualization of phylogenomic data. Mol Biol Evol 33:1635–1638. doi:10.1093/molbev/msw046. 26921390 PMC4868116

[93] Brautigam CA, Deka RK, Norgard MV 2020 Crystal structure of TP0037, a D-lactate dehydrogenase, from Treponema pallidum. RCSB PDB www.rcsb.org/structure/7JP2 (accession no. 7JP2). 10.1128/mBio.02249-20 PMC7512555 32963009

[94] Brautigam CA 2015 Calculations and publication-quality illustrations for analytical ultracentrifugation data. Methods Enzymol 562:109–134. doi:10.1016/bs.mie.2015.05.001. 26412649

