# Supplementary figures and images for: Biophysical and Biochemical Characterization of TP0037, a d-Lactate Dehydrogenase, Supports an Acetogenic Energy Conservation Pathway in Treponema pallidum
Source: mBio. 2020 Sep 22;11(5):e02249-20. doi: 10.1128/mBio.02249-20 (PMC7512555; doi:10.1128/mBio.02249-20)

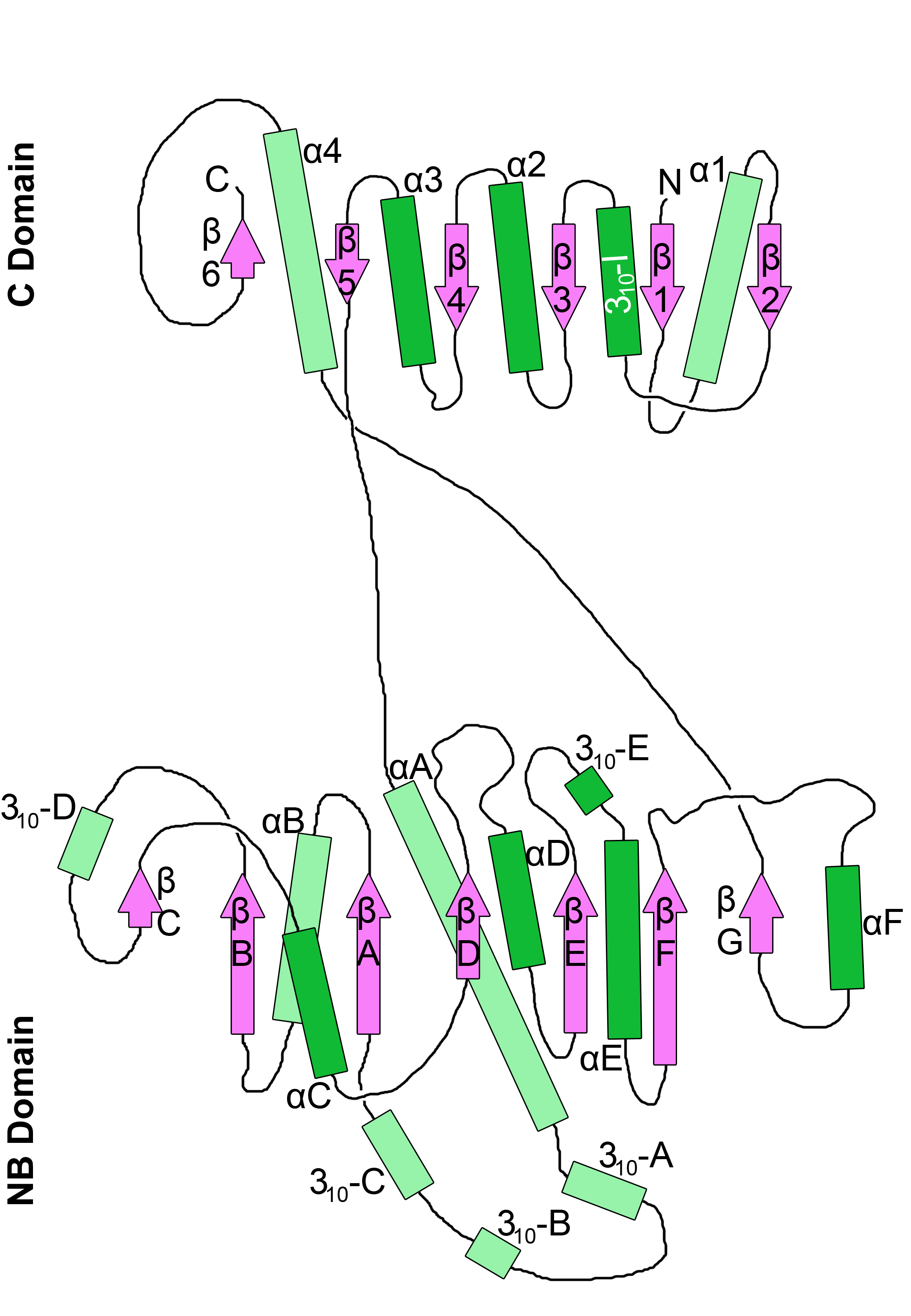

Supplement: FIG S1 [file mBio.02249-20-sf001.tif]
